# Effect of Speciation Transformation of Cadmium (Cd) on P-Wave Velocity Under Moisture Regulation in Soils

**DOI:** 10.3390/ma18020416

**Published:** 2025-01-17

**Authors:** Jun Fu, Han Zhou, Yanjin Luo, Bian Huang, Zixuan Qing, Ke Yan, Ying Shi

**Affiliations:** 1 Department of Geotechnical Engineering, Tongji University, Shanghai 200092, China; 2 Kunming Prospecting Design Institute of China Nonferrous Metals Industry Co., Ltd., Kunming 650051, China; 3 School of Resources and Safety Engineering, Central South University, Changsha 410083, China

**Keywords:** P-wave velocity, Cd-contaminated soil, soil porosity, heavy metal distribution

## Abstract

This study aims to investigate the influence of cadmium (Cd) speciation transformation on P-wave velocity under different soil moisture conditions, providing critical insights into the subsurface characteristics of contaminated soils. Taking Cd-contaminated soil as the research subject, P-wave velocity and the speciation distribution of Cd in soils with different moisture contents and Cd adsorption levels were measured. The results reveal that when the soil is contaminated by Cd, the porosity is altered and it eventually lead to change P-wave velocity. By increasing the moisture content of soils, the redox potential (Eh) rises and the pH decreases, which lead to the speciation transformation of Cd from carbonate-bound state (CAB), Fe-Mn oxide-bound state (FMO), and organic and sulfide-bound state (ORB) to the exchangeable state (EX). These transformations of Cd to EX result in the increase in soil porosity, which lead to the decrease in P-wave velocity. In addition, linear regression analysis was conducted the P-wave velocity (∆*V*) and the EX (∆*EX*) at various Cd adsorption levels. The analysis shows that there is a strong linear relationship between exchangeable Cd content and P-wave velocity, and the determination coefficient is about 0.9, which provides a reliable basis for monitoring soil Cd contamination by using P-wave velocity. This study provides valuable insights into the relationship between the speciation distribution of heavy metals in soil and the properties of acoustic wave.

## 1. Introduction

Geophysical exploration plays a critical role in understanding the subsurface characteristics of soils, employing techniques such as gravity, magnetism, electrical resistance, and seismic waves. Among these, seismic waves, particularly P-waves, stand out due to their efficiency, speed, and non-intrusive nature. P-waves are longitudinal waves that travel through the Earth’s subsurface, providing valuable information about the physical properties of soil. For example, Ji et al. [1] used acoustic wave velocity to determine the thermal conductivity of frozen clay soil, while Kurtulus et al. [2] employed P-wave velocity to estimate the Atterberg limit and bulk mass density of expansive soils. The use of P-waves in soil analyses offers a non-destructive method for assessing soil characteristics in various environmental and engineering applications.

The velocity of P-waves through soil is influenced by several factors, with soil porosity being one of the most significant. Numerous studies have documented a negative correlation between P-wave velocity and soil porosity [3,4,5,6]. For instance, when clay is added to granular materials, it tends to fill the pores, thereby increasing the P-wave velocity, as observed by Minear [5]. This phenomenon occurs because elastic waves primarily propagate through the solid skeleton of the soil, and as the wave encounters pores, it undergoes refraction and scattering, leading to energy attenuation and a reduction in wave speed. Therefore, changes in soil porosity directly affect the speed at which P-waves travel through the soil, making P-wave velocity a useful indicator of soil porosity. Researchers have also explored the quantitative relationship between P-wave velocity and soil porosity, developing empirical models to predict porosity changes based on P-wave velocity measurements. For example, Shen et al. [7] established a relationship between P-wave velocity and porosity for soils of varying thicknesses, and this model accurately depicted porosity variations in different soil layers. Similarly, Uyanık [8] conducted experiments to derive an equation linking P-wave velocity with porosity in shallow clay soils, achieving a high correlation coefficient of approximately 0.99. These studies underscore the potential of using P-wave velocity as a reliable, quantitative tool to monitor changes in soil porosity.

The existence of some soil factors, such as organic matter or heavy metals, can change the pore structure and thus change the P wave velocity. In recent years, heavy metal contamination of soils has become a significant environmental concern worldwide. Heavy metals, including cadmium (Cd), lead, arsenic, and mercury, can accumulate in soils due to industrial activities, agricultural practices, and urbanization. These contaminants pose serious risks to human health, ecosystems, and agricultural productivity. Understanding how heavy metals interact with soil components and affect soil properties is crucial for assessing environmental risks and developing effective remediation strategies.

The influence of heavy metals on P-wave velocity has been investigated in previous studies, with researchers focusing primarily on the total heavy metal content in soils. Luo et al. [9] examined the effect of the heavy metal content on P-wave velocity, finding that the adsorption of heavy metals by soil particles leads to changes in porosity, which, in turn, affects the velocity of P-waves. However, the impact of heavy metals on soil properties is not solely determined by their total concentrations. The speciation distribution of heavy metals within the soil—whether they are bound to organic matter, oxides, or present in exchangeable forms—also plays a crucial role in determining the extent of contamination and its effects on soil behavior. In soils contaminated with heavy metals, various processes such as diffusion, redox reactions, and chelation influence the speciation distribution of these metals between the solid and liquid phases. There are limited studies on how the speciation transformation of these heavy metal forms affects the properties of soil, especially the soil porosity, thus affecting the P-wave velocity.

The research utilizes P-wave monitoring to assess how changes in soil porosity, induced by Cd contamination, influence P-wave velocity. Specifically, the study examines how the transformation of Cd from less mobile states (such as carbonate-bound, Fe-Mn oxide-bound, and organic or sulfide-bound forms) to the more mobile exchangeable state affects the soil porosity and, consequently, the P-wave velocity. By manipulating the soil moisture content, the study explores the dynamics of Cd transformation and its impact on soil acoustic properties. Then, through a linear fitting analysis, the relationship between Cd speciation transformation, soil porosity, and P-wave velocity is quantitatively assessed. The findings of this research provide new insights into the interactions between heavy metal contamination and soil acoustic properties, with significant implications for environmental monitoring and risk assessment. The ability to detect changes in soil porosity and heavy metal distribution through P-wave velocity measurements could offer a valuable tool for real-time ecological risk monitoring, helping to identify areas of concern and guide remediation efforts.

## 2. Materials and Methods

### 2.1. Sampling

**Figure 1:**
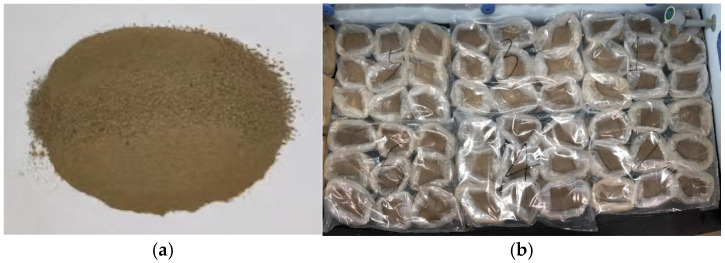
Soil samples: (**a**) in situ soil sample; (**b**) treated raw soil sample.

The soil samples used in this experiment were collected from the loamy soil in Changsha City, Hunan Province, at a depth of 0–3 m in a farming base layer (as shown in Figure 1a). The collected soil samples were initially pretreated by crushing large soil clumps and manually removing impurities such as residual crop roots and rubble (as shown in Figure 1b). The treated soil samples were then air-dried in a ventilated area. Once dried, the soil was further ground using a horizontal planetary mill (XQM-0.4L) (Changsha, China) and passed through a 2 mm soil sieve to serve as the raw material for preparing contaminated soil samples. The processed soil was stored in sealed bags for future use. The basic properties of the soil are shown in Table 1.

### 2.2. Preparation of Soil Samples with Different Cd Sorption Levels

Cadmium nitrate tetrahydrate (Cd (NO_3_)_2_·4H_2_O) was weighed using an electronic balance (Shanghai Precision Instrument Co., Ltd., Shanghai, China) and dissolved in water to prepare the Cd (NO_3_)_2_ solution of the desired concentration. After the solution was fully prepared, 600 g of soil was gradually added to the solution (maintaining a water: soil ratio of 1:2.2) while stirring at 300 rpm for 30 min to ensure thorough mixing. The mixture was then poured into a 50 × 50 × 50 mm^3^ mold, with continuous shaking to eliminate air bubbles, which could attenuate sound waves during soil penetration. The samples were maintained for 7–14 days, after which they were dried and cooled to room temperature, and then sealed in bags to prevent moisture absorption. The total Cd content in the soil was determined using the soil ablation method. Three parallel sets of samples were prepared for each concentration gradient to minimize random experimental errors. Cadmium adsorption gradient settings and doses of drugs added are shown in Table 2. 

### 2.3. Preparation of Soil Samples with Different Moisture Contents

Ultra-pure water was sprayed onto the surfaces of Cd-contaminated soil specimens with varying concentrations of heavy metals. The treated samples were sealed in airtight bags and allowed to sit for approximately 10 days to ensure uniform moisture distribution. This process was repeated eight times to reach the desired moisture content. After each iteration, the actual moisture content was measured using the standard GB7172-1987 method. Table 3 provides the details of the water mass required and the corresponding measured moisture content.

### 2.4. Acoustic Measurement Method

**Figure 2:**
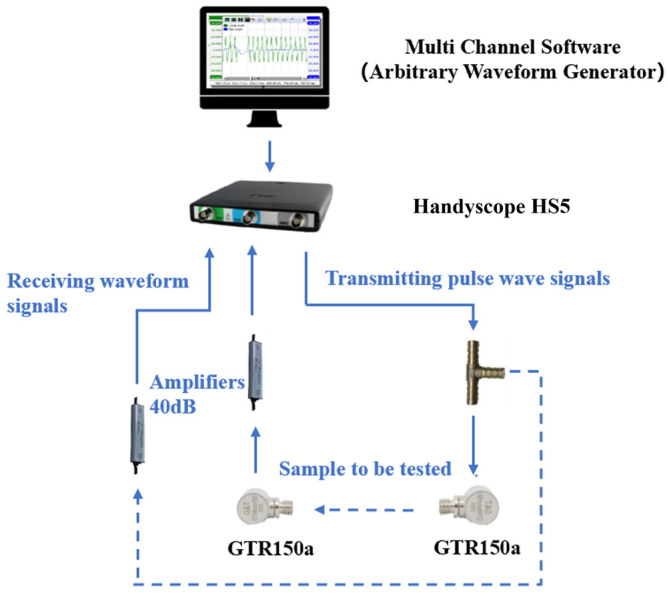
Combined acoustic system.

**Figure 3:**
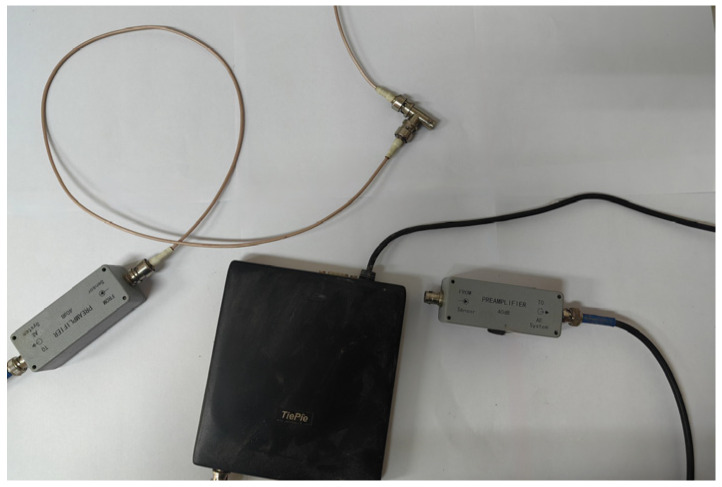
Practical operation of the combined acoustic wave test system.

As illustrated in Figure 2, the P-wave velocity of loamy soils was measured using a combined acoustic monitoring system, which included TiePie Multi Channel oscilloscope software, a TiePie engineering instrument (Handyscope HS5, TiePie Engineering, Sneek, The Netherlands), and an acoustic emission sensor (GTR150a, Hunan Enditi Technology Co, Ltd., Changsha, China). The TiePie instrument emitted a fixed pulse wave signal with a frequency of 10 Hz and a pulse width of 5 μs. This signal was amplified by 40 dB and propagated through the soil sample, with the results displayed on the multi-channel software. The measurement time was recorded from the VALUE window of the software. To account for zero error, the probes of the acoustic emission sensor were connected, allowing for the real propagation time of the P-wave through the soil sample to be calculated by subtracting the zero error from the measured time. The distance between the probe surfaces was measured using a Vernier caliper, and the P-wave velocity was determined by dividing this distance by the real propagation time. The test procedure is depicted in Figure 3.

### 2.5. Eh and pH Determination Methods

The soil pH was measured using an electrode in accordance with ISO 10390:2021 [9]. The electrode was immersed in a prepared soil sample solution, and the pH value was recorded once the reading stabilized. To enhance accuracy, the measurements were repeated. The redox potential (Eh) of the soil was determined using the HLY-216 redox potential tester (HanlinYuan Technology Co., Ltd, Wuhan, China). The actual soil Eh value was calculated by adding the measured value to the redox potential of the saturated glycolic electrode at the same temperature. For soils with low initial moisture content, ultra-pure water was applied to the soil surface to ensure close contact with the polarization electrode during measurement.

### 2.6. Extraction Methods of Heavy Metal Forms

The Tessier sequential extraction method was employed to isolate each heavy metal form, as detailed in a previous study [10]. The supernatants from all extracted fractions were analyzed for heavy metal concentrations using inductively coupled plasma mass spectrometry (ICP-MS) (PerkinElmer, Hopkinton, MA, USA).

## 3. Results and Discussions

### 3.1. Effect of Soil Moisture Content on P-Wave Velocity

**Figure 4:**
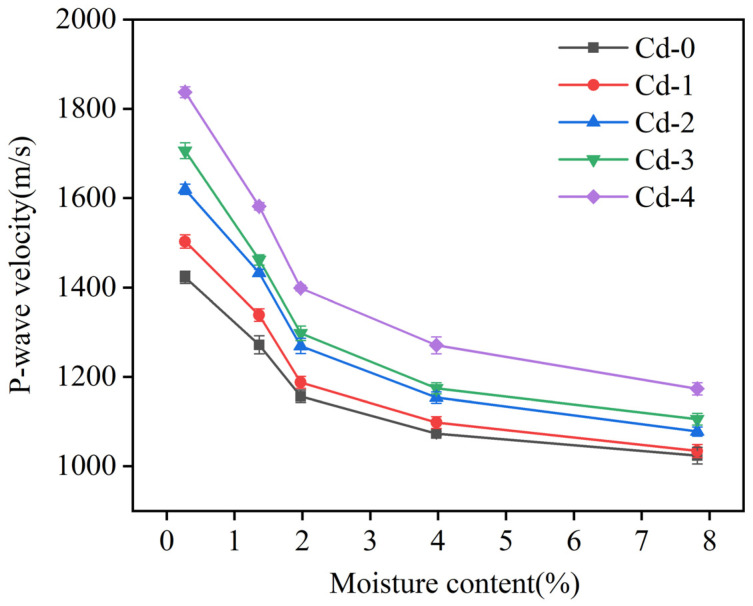
Relationship between soil moisture content and P-wave velocity at different Cd adsorption levels.

The P-wave velocity of soils with different moisture contents and different Cd adsorption levels was measured. As shown in Figure 4, the P-wave velocity was sensitive to changes in the soil moisture content. Across all levels of Cd adsorption, an increase in the soil moisture content corresponded with a decrease in the P-wave velocity. In the Cd-0 group (with a Cd adsorption level of 0.21 mg/kg), increasing the soil moisture content from 0.27% to 7.82% resulted in a reduction in the P-wave velocity from 1422.87 m/s to 1023.86 m/s. This pattern of decline was consistent across soils with varying Cd adsorption levels.

Overall, as the soil moisture content rose from 0.27% to 7.82%, the P-wave velocity decreased at an average rate of approximately 38.6%. This reduction aligns with the findings of Sharma and Gupta [11], who also observed that a rise in moisture content leads to a decline in P-wave velocity across various soil types. In soils with a low moisture content, drying typically enhances the soil’s capacity to adsorb moisture. This causes the soil particles to bind more closely together, reducing the volume of pore spaces, also known as the soil porosity. As a result, when the soil is dry or contains only minimal moisture, the solid structure of the soil matrix is predominant, allowing P-waves to travel faster due to the dense packing of soil particles. In this condition, P-waves can propagate through the solid particles with minimal energy loss, leading to a higher P-wave velocity [12].

Water in soil exists primarily in two forms: bound water and free water. Bound water refers to water molecules that tightly adhere to soil particles, typically forming a thin film around them. This type of water is immobile and does not flow freely through the soil. As bound water content increases, the film becomes thicker, effectively enlarging the size of the soil particles. This enlargement leads to a greater overall soil porosity because the particles are separated by a thicker layer of water. Conversely, free water refers to the water that can flow through the soil pores under the influence of gravity. Free water occupies the larger voids between soil particles and, as its content increases, so does the hydrodynamic pressure within the soil. This increase in pressure can further expand soil pore spaces, which also contributes to a higher porosity. The combined effect of both forms of water is an increase in soil porosity, which reduces the medium’s ability to transmit seismic waves efficiently, resulting in a decrease in P-wave velocity [13]. Thus, as moisture content increases, the structure of the soil becomes less consolidated, causing a significant reduction in the speed at which P-waves propagate.

It is important to note that the rate of P-wave velocity decrease is not constant. The rate of decline is more pronounced at lower moisture levels and slows as the moisture content continues to rise. When the soil moisture content is low, water is mainly present in the form of bound water, which has a greater impact on soil structure than free water. Bound water disrupts the original crystalline structure of the soil, increasing the spacing between soil particles and weakening the intermolecular forces that normally hold the soil together. This disruption leads to significant changes in soil porosity, causing a sharp decrease in P-wave velocity. As the moisture content rises and bound water reaches its saturation point, any additional water transitions into free water, which has a different impact on the soil structure [13].

Free water primarily moves through soil pores due to the force of gravity, filling larger pore spaces and creating fewer changes in the crystal structure of the soil. This difference in the roles of bound and free water explains why the rate of decrease in P-wave velocity slows as the moisture content continues to increase. When free water dominates, its effect on soil structure is less dramatic than that of bound water. Free water mainly influences the larger voids in the soil, whereas bound water impacts the microstructure by altering the particle-to-particle interactions. Consequently, the reduction in P-wave velocity becomes less pronounced at higher moisture levels because the additional water content mainly fills the existing pore spaces rather than further breaking down the solid structure of the soil. This distinction between the effects of bound water and free water helps to clarify the complex relationship between soil moisture content, soil porosity, and P-wave velocity [14].

### 3.2. Variation in P-Wave Velocity with Cd Concentration

**Figure 5:**
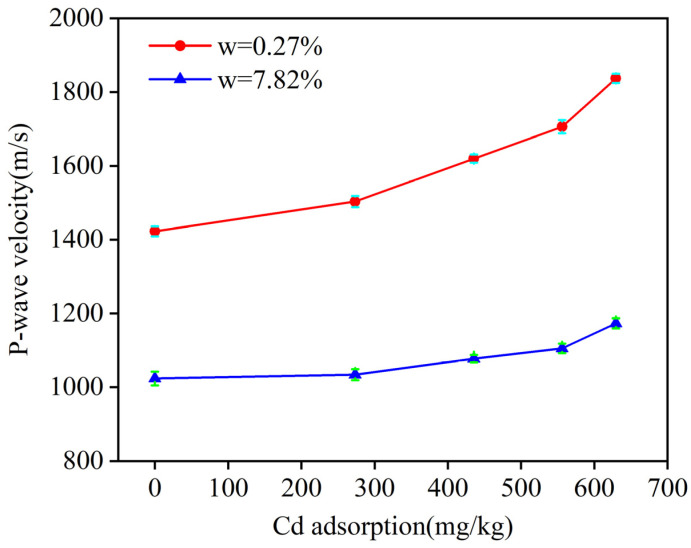
Relationship between soil Cd adsorption and P-wave velocity at moisture contents of 0.27% and 7.82%.

Under the same moisture content, the P-wave velocity of soil samples with different Cd adsorption levels is quite different. To determine whether P-wave velocity is influenced by Cd adsorption, Figure 5 illustrates the relationship between P-wave velocity and Cd adsorption at moisture contents of 0.27% and 7.82%.

As shown in Figure 5, the P-wave velocity increases consistently with a higher Cd adsorption. At the moisture content of 0.27%, when the Cd adsorption increased from 0.21 mg/kg to 629.43 mg/kg, the P-wave velocity rose markedly from 1422.87 m/s to 1837.15 m/s. Similarly, at the moisture content of 7.82%, an increase in Cd adsorption from 0.21 mg/kg to 629.43 mg/kg led to a rise in P-wave velocity from 1023.86 m/s to 1173.27 m/s. These increases in P-wave velocity can be attributed to the changes in Cd adsorption within the soil. When introduced into the soil, Cd is adsorbed onto the colloidal surface through electrostatic interactions. This interaction leads to the formation of macromolecular particles as colloids and metal ions combine, filling the soil pores and reducing the soil porosity, which in turn increases the P-wave velocity [15].

**Figure 6:**
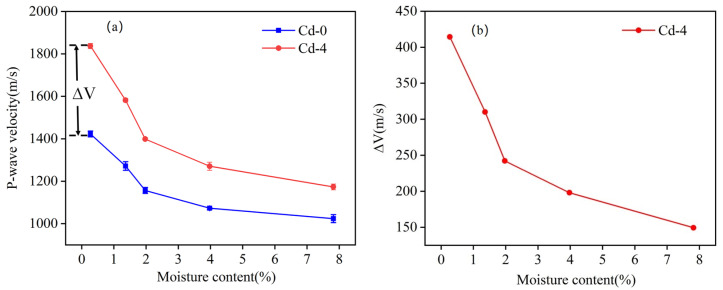
(**a**) Schematic of Δ*V*; (**b**) Variation in Δ*V* with moisture content.

To gain a deeper understanding of how the P-wave velocity changes, the study uses Cd-0 as a baseline to calculate the change rate of the P-wave velocity under the specific moisture content. The change rate in the P-wave velocity at the same moisture content is defined as follows:

(1) $$\Delta V_{x}{=V}_{\mathrm{Cd}\text{-}x}{\;-\;V}_{\mathrm{Cd}\text{-}0}$$

where $\Delta V_{\mathrm{Cd}\text{-}x}$ represents the P-wave velocity at the Cd-x level, and $\Delta V_{\mathrm{Cd}\text{-}0}$ represents the P-wave velocity at the Cd-0 level. Figure 6a displays the Δ*V*_4_ when the Cd adsorption level is 629.43 mg/kg (Cd-4).

The variation in Δ*V_4_* with different moisture contents is shown in Figure 6b, representing the change in P-wave velocity caused by Cd adsorption at a specific moisture content. If the effects of the moisture content and Cd adsorption on the P-wave velocity were independent, Δ*V*_4_ would remain constant at a specific Cd adsorption level. However, Δ*V*_4_ decreases as the moisture content increases. For example, at a moisture content of 0.27%, Δ*V*_4_ was approximately 414.27 m/s. As the moisture content increased, Δ*V* gradually decreased, reaching only 149.41 m/s at a moisture content of 7.82%. Based on the reduction in Δ*V*_4_ as shown in Figure 6b, it can be inferred that the moisture content and Cd adsorption levels have a coupling effect on the P-wave velocity.

Actually, the interaction between moisture content and Cd adsorption levels has been studied [16,17]. Upon entering the soil, Cd binds with components such as carbonate, organic matter, and iron-manganese oxides in the soil through diffusion, adsorption, and complexation. Consequently, Cd is distributed in different forms in the soil and its solution. According to the Tessier extraction method, Cd in soil or sediment can be divided into five forms: the exchangeable state (EX, including the water-soluble state), carbonate-bound state (CAB), Fe-Mn oxide-bound state (FMO), organic and sulfide-bound state (ORB), and residual state (RES) [10]. Various forms of Cd may undergo transformations among each other, eventually forming a dynamic equilibrium. This dynamic equilibrium is influenced by some factors, such as Eh, pH, carbonate, organic matter, and iron-manganese oxide contents [18,19]. In this study, changes in moisture content can break the original dynamic equilibrium, consequently prompting the speciation transformation of Cd. This transformation alters the solid/liquid distribution of Cd, potentially influencing the porosity of the soil [20]. Subsequently, the propagation of P-waves in the soil is affected, leading to changes in P-wave velocity [21]. Therefore, it can be inferred that under the same Cd content, the speciation transformation of Cd due to the moisture content might be related to the difference in the Δ*V*.

### 3.3. Coupled Effect of Soil Moisture Content and Cd Adsorption Level on P-Wave Velocity

Based on the speculation above, a further analysis is needed to investigate how the coupled effect of Cd adsorption level and moisture content affect the speciation transformation of Cd. The physicochemical properties of the soil (Eh and pH) were measured with different moisture contents. Subsequently, the speciation changes in Cd were evaluated under varying conditions of moisture content and Cd adsorption levels, aiming to elucidate the underlying mechanisms driving these transformations.

#### 3.3.1. Speciation Transformation of Cd with Different Moisture Contents

**Figure 7:**
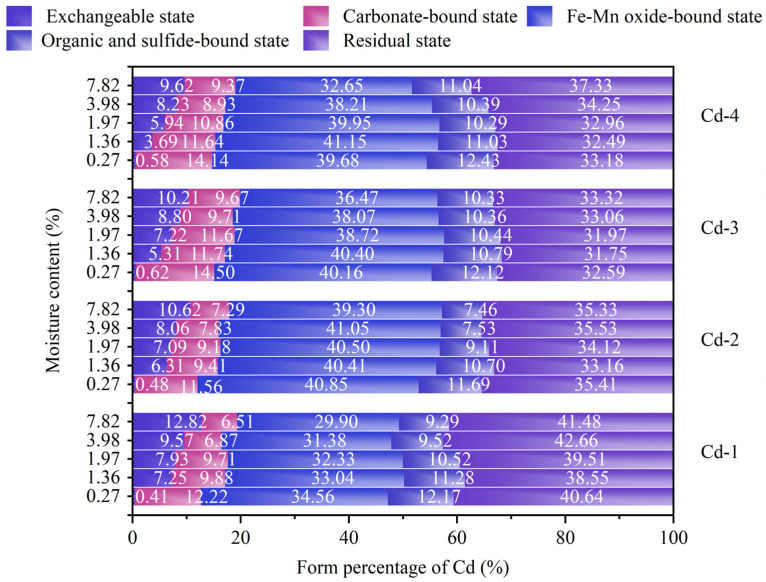
Distributions of Cd forms at different moisture contents.

The properties of the five forms of heavy metals divided based on the Tessier extraction method are shown as in Figure 7. EX is adsorbed on the surface of soil under the action of Coulomb force, has a high level of activity, and is easily released into the free state [22,23]. CAB is formed by the co-precipitation of heavy metals with carbonate in soil, which is sensitive to soil pH [24]. FMO binds through strong ionic bonds, reacting with iron-manganese oxides to form complexes or encrusting on the surface of precipitate particles, which is sensitive to soil pH and Eh [25]. ORB is formed by the chelation of heavy metals with organic matter in the soil or exists in the form of sulfides, mainly influenced by soil Eh [26]. RES generally exists in the mineral lattice of the soil and is not easily released under natural conditions [27].

In order to analyze the effect of the moisture content on the speciation transformation of Cd, the distributions of Cd forms at different moisture contents were measured. As shown in Figure 7, no matter what the adsorption level of Cd was, as the water content increased, RES remained relatively stable, FMO, CAB, and ORB showed a downward trend, and EX showed an increasing trend. Taking group Cd-1 as an example, within the moisture content range of 0.2% to 7.8%, FMO decreased from 34% to 29%, CAB decreased from 12% to 6%, and ORB decreased from 13% to 9%, while EX increased from 0 to 14% and RES remained almost unchanged at about 40~41%. The results show that the increase in moisture content promotes the transformation of Cd from FMO, CAB, and ORB to EX.

#### 3.3.2. Evolution of pH and Eh of Soil with Different Moisture Contents

**Figure 8:**
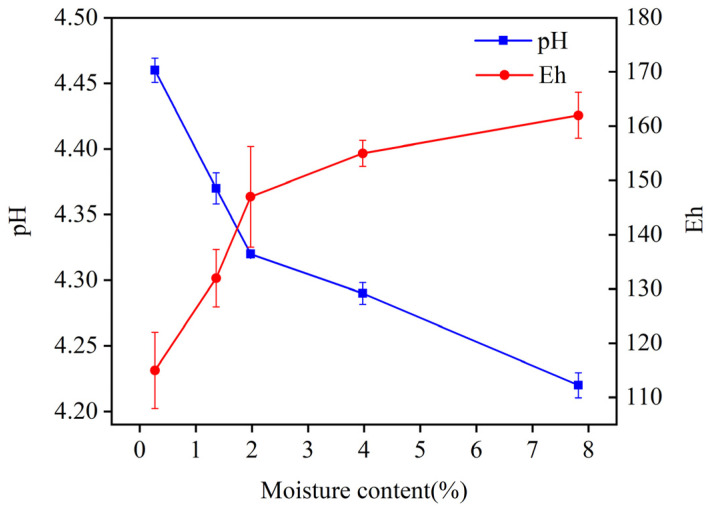
Change in Eh and pH with soil moisture content in group Cd-1.

Moisture content can alter the physicochemical properties of the soil, such as Eh and pH. As shown in Figure 8, Eh and pH varied with the soil moisture content when the Cd adsorption level was 273.29 mg/kg. As the moisture content increased, Eh rose and pH decreased. Specifically, Eh increased from 115 mV to 162 mV, showing an increase of 40.87%. And pH decreased from 4.46 to 4.22, indicating a decrease of 5.38%. It is shown that changes in moisture content cause changes in the Eh of soil, leading to variations in the solubility of heavy metals and consequent changes in the EX content [28]. A change in pH alters the adsorption capacity of the soil for heavy metals, promoting a speciation transformation [28,29]. As a result, the changes in pH and Eh affected the solubility of heavy metal complexes and triggered redox reactions within these complexes, subsequently affecting the soil’s adsorption capacity for heavy metals [30,31].

#### 3.3.3. Relationship Between pH and Eh and Ex Form of Cd in Soil

To quantify the relationships between pH and Eh changes and the various forms of Cd in the soils, a linear fit was employed, and the results are presented in Table 4.

The data in Table 4 reveal a highly significant negative correlation between pH and EX contents. This finding aligns with previous research indicating that a decrease in pH typically leads to an increase in exchangeable Cd [32,33,34,35]. In the soil, the mechanisms by which pH affects EX fall into three main categories. Firstly, pH affects the solubility of carbonate precipitates, iron, and manganese oxides. Under acidic conditions (a low pH), carbonate precipitates and Fe-Mn oxides dissolve and lead to the release of Cd absorbed by these compounds into an exchangeable state and thus to an increase in EX [32,33]. Secondly, pH affects the charged electric particles on the surface of soil particles. Under acidic conditions, the higher number of positive charges on the surface of soil particles restricts the adsorption of Cd^2+^, due to homogeneous repulsion, resulting in an increase in exchangeable Cd [34]. Thirdly, pH affects the Cd adsorption capacity of the soil. Under acidic conditions, Cd^2+^, which is electrostatically adsorbed on the surface of soil minerals or organic matter, is easily displaced by the large amount of H^+^ hydrolyzed from the soil, leading to an increase in EX [35]. In contrast, Eh showed a highly significant positive correlation with EX, mainly by facilitating the conversion of ORB to EX. Frohne et al. [36] pointed out that the high redox potential tends to oxidize the sulfide to sulphate, releasing the heavy metals originally bound to the sulfides in large quantities. This resulted in lower ORB contents and higher EX contents.

#### 3.3.4. Relationship Between P-Wave Velocity and EX Contents

It has been concluded that with the increase in moisture content, Cd undergoes a transformation from CAB, FMO, and ORB to EX. To further explore the relationship between the change degree of speciation transformation and the change degree of P-wave velocity, Δ*EX_x_* was introduced, showing the content of EX increased with increases in the moisture content.

**Figure 9:**
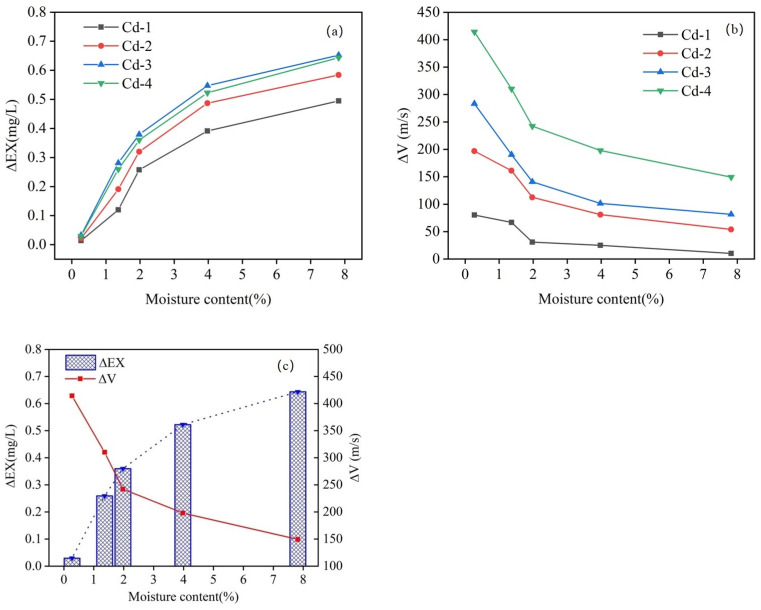
Δ*EX_x_* (**a**) and Δ*V_x_* (**b**) with different moisture contents; (**c**) group Cd-4 as an example.

Figure 9 illustrates the changes in Δ*EX_x_* and Δ*V_x_* across different moisture content levels. Figure 9c shows group Cd-4 as an example; as the moisture content increased, Δ*EX_x_* experienced a rapid initial rise, followed by a gradual slowing of the rate of increase. Simultaneously, Δ*V*_4_ displayed a rapid initial decrease, followed by a gradual slowing of the rate of decrease. Specifically, in the 0.27–1.97% moisture content interval, Δ*EX*_4_ increased rapidly from 0.03 mg/L to 0.36 mg/L, with a slope of 0.19. During this phase, Δ*V*_4_ decreased rapidly from 414.27 m/s to 242.17 m/s, with a slope of −97.79 m/s. In the moisture content interval from 1.97% to 7.82%, Δ*EX*_4_ increased at a slower rate, from 0.36 mg/L to 0.64 mg/L, with a slope of 0.05. Correspondingly, Δ*V*_4_ decreased at a slower rate, from 242.17 m/s to 149.41 m/s, with a slope of 15.86. This indicates a corresponding relationship between them. That is, Δ*V*_4_ decreases as Δ*EX*_4_ increases.

**Figure 10:**
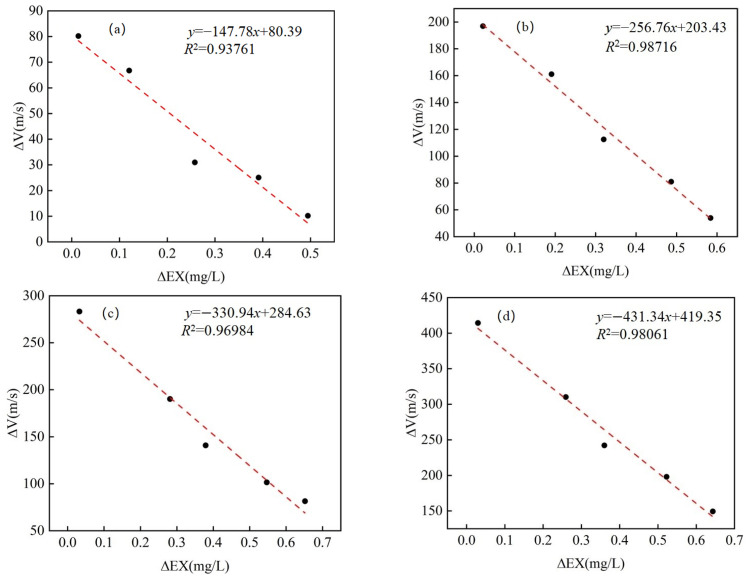
Linear fit of Δ*EX_x_* versus Δ*V_x_* for all groups. (**a**) Group Cd-1; (**b**) Group Cd-2; (**c**) Group Cd-3; (**d**) Group Cd-4.

To explore the relationship between Δ*V_x_* and Δ*EX_x_*, a linear regression analysis was conducted for different Cd adsorption levels. As shown in Figure 10, fit functions had high coefficients of determination (R^2^ > 0.9) for all Cd adsorption levels, indicating a significant negative correlation between Δ*V_x_* and Δ*EX_x_*. This result provides theoretical possibilities for employing P-waves to detect microscopic speciation transformations of heavy metals in the soils. Previous studies have indicated that the velocity of P-waves in soil is influenced by the concentration of Cd [8]. This study further refines the analysis to reveal that the velocity of P-waves is jointly affected by both the concentration of Cd and the soil moisture content. The results demonstrate a linear correlation between the variations in heavy metal content, specifically that of Cd, and the velocity of P-waves. This research provides evidence for the potential use of P-wave velocity for the in situ monitoring of heavy metal pollution in soils.

**Figure 11:**
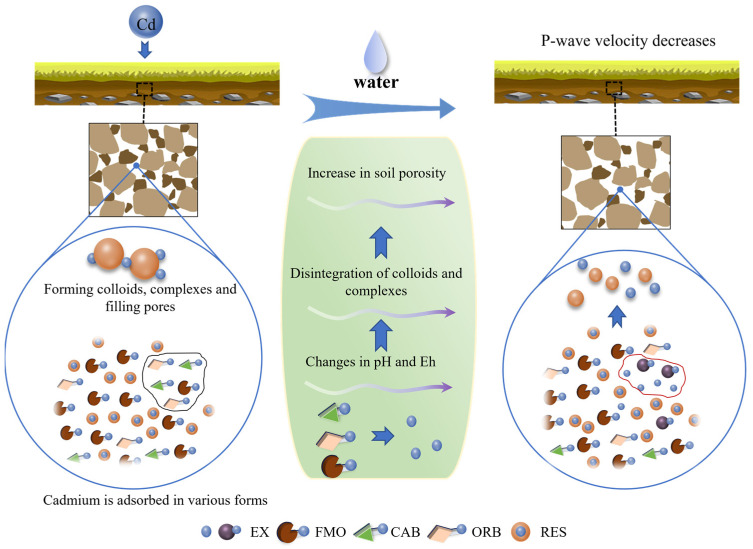
Schematic representation of speciation transformation of Cd, which affects P-wave velocity.

The results above indicate that the transformation of Cd forms under water regulation results in changes in P-wave velocity. A schematic diagram, as shown in Figure 11, is constructed to illustrate how the speciation transformation of Cd affects the P-wave velocity.

For Cd-contaminated soils, when Cd is adsorbed by the soil in different forms, it forms colloids and complexes. These substances then coalesce to form microaggregates, filling the soil pores and resulting in a decrease in soil porosity. As a result of this process, the P-wave velocity increases with the Cd adsorption [3,4]. As the water is added into the soil, both free water and bounded water contribute to the increase in soil porosity, resulting in a decrease in P-wave velocity. Under the coupled effect of moisture and Cd content, changes occur in pH and Eh. The higher the moisture content, the more intensive the transformation of Cd from FMO, CAB, and ORB to EX. During the transformation process, the carbonate co-precipitates are dissolved, the Cd originally adsorbed on the surface of the Fe-Mn oxides is released, the Cd complexed with the sulfide precipitates is desorbed, and, therefore, the previously formed colloids and complexes are disintegrated. This leads to the replacement of the initially occupied soil pores with air or water, resulting in a larger soil porosity. Therefore, the increase in soil porosity that resulted from the conversion from other states to the exchangeable state of Cd leads to the decrease in P-wave velocity. In summary, when Cd enters into the soil, an increase in Cd adsorption leads to an increase in P-wave velocity. However, as the moisture content increases, the rise in water content results in a decrease in P-wave velocity, which weakens the increase in P-wave velocity due to the increase in Cd adsorption level.

## 4. Conclusions

The variation pattern of P-wave velocity was investigated under the coupled regulation of Cd adsorption level and moisture content, and the findings are as follows:

(1) P-wave velocity is found to decrease with increasing soil moisture content. However, under the same moisture change conditions, the reduction in P-wave velocity in Cd-contaminated soil is more pronounced than in nearly uncontaminated soil. This observation suggests that factors in addition to the moisture content significantly influence the P-wave velocity;
(2) An increase in soil moisture content facilitates the transformation of Cd from more stable forms (FMO, CAB, and ORB) to a less stable form (EX), thereby enhancing the ecological risk of Cd in the soil. The analysis of the transformation mechanism reveals that pH is highly significantly negatively correlated with the EX content, while Eh is highly significantly positively correlated with the EX content. These correlations indicate that pH and Eh changes are the primary drivers of Cd speciation transformation;
(3) A significant correspondence exists between the EX content and the relative values of the P-wave velocity. The linear regression analysis demonstrates that the coefficients of determination (R^2^) for the fitted equations are consistently above 0.9 for the same adsorption amount, indicating a strong linear relationship. This finding provides a theoretical basis for utilizing P-wave velocity as an indicator for monitoring the soil EX content under fluctuating external moisture conditions;
(4) The variation in P-wave velocity is influenced by the synergistic effects of moisture conditions and Cd levels in the soil. Changes in water content lead to the speciation transformation of Cd, causing the disintegration of pre-existing complexes and colloids and the disruption of microaggregates. These alterations result in increased soil porosity, which consequently contributes to a decrease in P-wave velocity.

## Figures and Tables

**Table 1:** Soil’s basic properties.

Parameters	pH	Moisture Content (%)	Density (g/cm^3^)	CEC (cmol(+)/kg)	Organic Matter (g/kg)	Calcium Carbonate (g/kg)	Manganese Oxides (g/kg)
soil sample	4.76	1.53	1.67	10.6	21.4	6.8	0.45

**Table 2:** Cd adsorption gradient settings and drug dosage.

Group	Cd-0	Cd-1	Cd-2	Cd-3	Cd-4
Expected Cd adsorption (mg/kg)	0	300	450	600	700
Added Cd (NO_3_)_2_·4H_2_O(g)	0	0.4940	0.7410	0.9880	1.1526
Measured Cd adsorption (mg/kg)	0.21	273.29	435.41	556.04	629.43

**Table 3:** Ultra-pure water masses required and measured moisture content.

Expected Moisture Content (%)	0	1	2	4	8
Ultra-pure water masses required (g)	0	0.505	1.14	2.09	3.98
Measured moisture content (%)	0.271	1.363	1.974	3.975	7.819

**Table 4:** Correlation analysis of soil Eh and pH with contents of Cd forms.

Groups	Parameter	EX	CAB	FMO	ORB
Cd-300	pH	−0.96664 **	0.92749 *	0.87717	0.93294 *
	Eh	0.97352 **	−0.93528 *	−0.88668 *	−0.88672 *
Cd-450	pH	−0.97804 **	0.92588 **	0.92051 *	0.92642 *
	Eh	0.98773 **	−0.97525 **	−0.89654 *	−0.89462 *
Cd-600	pH	−0.98955 **	0.94919 *	0.84412	0.97743 **
	Eh	0.98874 **	−0.95885 **	−0.8437	−0.99692 **
Cd-700	pH	−0.99149 **	0.96302 **	0.87504	0.98995 **
	Eh	0.98741 **	−0.97572 **	0.83406	−0.98382 **

where “*” means 0.01 < *p* < 0.05, indicating a significant result, and “**” means *p* < 0.01, indicating a highly significant result.

## Data Availability

The original contributions presented in this study are included in the article. Further inquiries can be directed to the corresponding author.
